# A national survey of clubs medical provision and facilities in BUCS American Football 2019–2020

**DOI:** 10.1007/s11845-022-03201-9

**Published:** 2022-11-21

**Authors:** Eleanor Louise Travis, Andrea Scott-Bell, Claire Thornton

**Affiliations:** 1 School of Health, Leeds Beckett University, Leeds, LS6 3QW UK; 2 Northumbria University, Newcastle-Upon-Tyne, UK

**Keywords:** American Football, British American Football, British Universities and Colleges Sport, Medical provision, Player welfare

## Abstract

**Background:**

British American Football (BAF) is a developing sport in the UK, with keen growth in the British Universities and Colleges Sport (BUCS) league. Participation in BAF carries risks and so to facilitate safe participation medical care services must be evaluated.

**Aims:**

To evaluate medical provision in BUCS American Football in the 2019–2020 season.

**Methods:**

An online survey tool was used to collect data from BUCS BAF teams in the 2019–2020 season. Thirty-one teams (from across England, Wales and Scotland) responded to questions on facilities, provision and procedures.

**Results:**

Almost 42% of teams had a regular team first aider who attended home games each week. Only 61.5% attended away games and 7.7% attended team training. Access to a first aider was not dependent upon division. Home games were more likely to be risk assessed and have an emergency action plan compared to away games. The majority of teams had access to automated external defibrillator (AED) within 100 m of the pitch, yet only 29% of staff were trained to use them. Almost 84% of teams reported carrying a designated fully charged phone (with signal). Prominent qualitative themes indicated were cost/funding as barriers to hiring qualified medical staff, lack of institutional support, unreliability of medical provision and inadequate facilities/preparation for games.

**Conclusions:**

These findings provide key information on the status of medical provision, facilities and protocols in BUCS BAF. Data reveals a lack of consistent medical personnel, particularly at training and away games, and training in emergency care.

## Introduction

The game of American Football (AF) is a growing sport in Great Britain with over 80 teams playing in the British Universities and Colleges Sport (BUCS) league, which is made up of approximately 4230 students [1]. BUCS is divided into four leagues: premiership, division 1, division 2 and associate league.

American Football is a collision sport that exposes athletes to both contact- and non-contact-related injuries [2], and is overseen by the British American Football Association (BAFA). Players are often subject to high-velocity movements and frequent collisions [3]. Tackling and blocking are fundamental features of the sport and this contact is responsible for a significant number of injuries [2]. Equally, many soft tissue injures are non-contact-related, yet are still prevalent in the sport [2, 4, 5].

The likelihood of sustaining a catastrophic injury in the game of British American Football (BAF) is currently unknown. However, injury rate comparisons have been made between BAF and US collegiate football [2]. British university players were found to be at greater risk of concussion and more severe injuries than US collegiate AF athletes, possibly due to a lack of strength, lack of player experience (the game is rarely played before university in the UK) or policing of illegal contact due to inexperienced officials [2]. Furthermore, there have been a number of publicly acknowledged fatal injuries in the British game within recent years [6–8].

Identifying injuries early is the first step to recovery and implementation of interventions [9, 10]. The national governing body (NGB) BAFA’s current medical policy states minimum guidelines for games.^1^ A summary of the 2022 medical policy minimum medical guidelines for tackle AF is highlighted in Table 1. BUCS 2019/20 guidelines across all sports advised that it was the responsibility of the host institution to ensure there was correct first aid cover for game days (regulation 9.3.1) [1]. Where there are no BUCS regulations, the rules of the sport’s NGB should be considered [1]. At present, Rugby union leagues that play under BUCS must adhere to regulation 4.3 which stipulates that a team must provide a first aid kit on match days only [12]. Unlike BAFA guidelines which are outlined in the British American Football Referees Association (BAFRA) rulebook, the BUCS rugby 15 s Super League Minimum Operating Standards (MOSs) provide guidelines for training as well as match days [12]. This document is provided directly to teams ensuring teams are notified of updated policy.

**Table 1:** Summary of the 2022 BAFRA medical policy for tackle AF

1.	There are three levels of healthcare practitioner: 1. Emergency first aider (a minimum level 3 first aid qualification is required) 2. First aid trained therapist (see point 2) 3. Immediate care practitioner (must meet the criteria for a first trained therapist and hold a qualification endorsed by the Faculty of Pre-Hospital Care) The minimum requirements vary depending on the level of the game.
2.	A first aid trained therapist is defined as a professional practitioner and graduate in appropriate discipline: (a) Doctor registered with the GMC (b) Nurse registered with the NMC (c) Physiotherapist registered with the HCPC (d) Paramedic registered with the HCPC (e) Sports rehabilitator registered with BASRaT (f) Sports therapist who is a full member of the Society of Sports Therapists, the Sports Therapy Association or the Sports Therapy Organisation (g) Sports massage therapist registered with the Sports Massage Association (h) Osteopath registered with the GOC (i) Chiropractor registered with the GCC
3.	The first aider should not be a team member.
4.	The number of first aiders required at each level of the game.
5.	A risk assessment should be carried out by the first aider.
6.	A “suitable” first aid kit which is approved by the first aider must be available.
7.	A telephone with signal and battery charge should be available.
8.	Medical facilities may not be shared by two opposing teams.
9.	It is the responsibility of the home team game management staff to ensure medical provision cover is met. Should the requirements not be met, the game will be suspended, and sanctions will apply to the home team.
10.	It is recommended that an AED be accessible in emergencies (ideally within 100 m of the pitch). There is no requirement for AED training for the first aider or other game day staff.
11.	An emergency first aider should be present as minimum provision during AF practice.

Potential issues regarding adequate pitch-side provision at grassroots level are currently an under-recognised problem and teams are likely to only be able to afford one medical practitioner [13]. Unlike their North American counterparts, many British Universities do not have the sport science or specialist sports medicine support for their athletes [14], despite many being able to afford these services [15]. Student-athlete experiences differ greatly and are dependent on the university they attend [16]. Some athletes may seek medical guidance in the local community from practitioners who do not have specialist sport training [14]. BUCS rugby union league has comparable injury risk to that of English Premiership Rugby [17]; therefore, it could be argued that similar injury profiles may occur in other sports at premiership level and universities should fund medical provision for these sports teams.

To facilitate safe participation, current medical care services must be evaluated. The aim of this study is to evaluate the medical provision and facility and equipment access in BUCS American Football and to make recommendations about safe participation and appropriate medical care.

## Methods

A survey comprising of 24 key questions relating to (i) medical personnel, (ii) medical facilities and equipment and (iii) confidence in emergency situations was distributed using the website OnlineSurveys. The survey was shared via social media platforms including Facebook, Twitter and Instagram between June 2020 and August 2020. The survey was completed by the club representative deemed most appropriate, e.g. team manager, head coach or first aider. Prior to answering the questionnaire, participants were required to read the participant information and indicate their consent. Ethical approval for this study was granted by a British University Ethics Committee.

Thirty-one of 80 teams completed the survey across the league (38% response rate): premiership (19.4%, *n* = 6), division 1 (35.5%, *n* = 11) and division 2 (45.2%, *n* = 14). All respondents played full contact American Football in the British Universities and Colleges Sport (BUCS) leagues.

Data analysis was performed in Excel Version 2102 (2016) (Microsoft Corporation Washington, USA) and SPSS Version 26 (IMB, Chicago, Illinios, USA). Descriptive statistics were calculated, and a Pearson’s chi-squared test was conducted to assess the relationship between division and access to a regular team medic. The alpha level was set at *p* < 0.05.

Open ended, optional commentary sections were included at the end of each section to allow participants the opportunity to expand upon their answers in previous questions. The response rate for the three commentary sections was 45%. A thematic analysis was conducted on the most recurrent and prominent themes across these sections to highlight similarities and differences in the insights of participants [18]. A summary of these qualitative comments helped further understand and explain some quantitative results in the discussion.

## Results

### Medical personnel/provision

A range of medical personnel attended games including physiotherapists (*n* = 10), graduate sports therapists (*n* = 2), paramedics (*n* = 13), St Johns Ambulance First aiders (*n* = 1), a student in training (*n* = 1) and a sports rehabilitator (*n* = 1). No doctors were present as pitch-side first aiders.

Less than half of teams (41.9%, *n* = 13) reported that they had a regular team first aider who attended each week. Of all the regular team first aiders, 100% (*n* = 13) were reported to attend home games in the 2019/2020 season and 61.5% attended away games. However, 61.5% (*n* = 8) of the regular team first aiders did not attend team training. Pearson’s chi square test found no relationship between divisions and whether teams had a regular team first aider or not (*p* =  − 0.127). When participants were asked the highest qualification the team first aider held, in most cases, this was unknown (61.3%, *n* = 19), followed by postgraduates (25.8%, *n* = 8).

Of those teams who did not have a regular team first aider who attended training (61.5%, *n* = 8), medical provision was provided by a second party; a coach with first aid training (54.8%, *n* = 17), player with first aid training (32.3%, *n* = 10), reported ‘other’ (12.9%, *n* = 4) and an external paramedic’s company (9.7%, *n* = 3). Those teams, who stated ‘other’, reported relying on university facility/ground staff who were first aid trained, students studying physiotherapy and coaches who had first aid training, but the level of first aid training was not stated.

A thematic analysis highlighted prominent themes. Several barriers to medical provision were discussed, including expense of hiring medical provision, the hiring of medical personnel with a lack of reliability and/or experience of the game of American Football and lack of institutional support. However, two individual teams commented that they were successful in hiring an external party to provide medical cover.

### Medical facilities

The range of medical equipment and facilities available is summarised in Table (2).

Further findings on AED access in Table 2, the majority of teams, 74.2% (*n* = 23), reported their home game facility had access to an AED within 100 m of the pitch and 80.6% (*n* = 25) reported their training facility had access to an AED within 100 m of the pitch.

**Table 2:** The range of medical equipment and facilities available

	Premiership (*n* = , %)	Division 1 (*n* = , %)	Division 2 (*n* = , %) *percentages out of total number in the division	Average (*n* = , %)
*Medical facilities*				
Designated fully charged phone (with signal) available at each game for emergencies	6 (100)	11 (100)	9 (64.2)	26 (83.9)
Designated medical room	2 (33.3)	8 (72.7)	2 (14.2)	12 (38.7)
Clear vehicular access for an ambulance or other emergency vehicle at the facility	6 (100)	10 (90.9)	14 (100)	30 (96.8)
Access to automated external defibrillator (AED)	6 (100)	5 (45.4)	5 (35.7)	16 (51.6)

^*^Brackets denote percentage of responses in each category

When asked if all game-day staff were trained to use an AED, only 29% (*n* = 9) reported ‘yes’. Of those participants who reported ‘no’, they noted that it was the site manager, general manager or specific coaching staff member who had this training.

Only 83.9% (*n* = 26) of teams reported carrying a designated fully charged phone (with signal) which was available at each game. Of those who answered ‘yes’ to carrying this phone, 65.4% (*n* = 17) reported the coach as carrying this phone, followed by the medic (15.4%, *n* = 4), game-day staff member (15.4%, *n* = 4) and unknown (3.8%, *n* = 1).

When asked if the team had a designated medical room at their home grounds (not including a change room facility or use of a spare changing room), 61.3% reported they did not.

The majority of teams (96.8%, *n* = 30) reported that there was clear vehicular access for an emergency vehicle at the facility, with 76.7% (*n* = 23) of those respondents indicating this was with direct access to the field of play.

### Policies/procedures and documentations

When asked about whether an emergency action plan (EAP), including a risk assessment, was outlined for each home game, 67.7% (*n* = 21) of all teams reported yes. However, fewer clubs had these documents in place for each away game, with 54.8% (*n* = 17) reporting they had no EAP in place for each away game.

Only 33.3% (*n* = 11) coaching staff reported feeling confident removing a player’s helmet and pads in an emergency, and 6% (*n* = 2) were not confident at all. Participants also reported on the confidence of their first aider to remove a player’s helmet and pads in an emergency. A total of 27.2% (*n* = 9) reported feeling very confident, and 3% (*n* = 1) not confident at all.

### Thematic analysis

The most recurrent themes across the commentary sections for medical personnel/provision and medical facilities are outlined next and in Table 3. Medical personnel/provision included cost/funding, lack of institutional support, unreliability, lack of experience in the game and lack of knowledge of the game. With regard to facilities, teams reported institutional barriers, lack of direct access and unknown equipment carried by the first aider.

**Table 3:** The most recurrent themes across the commentary sections for medical personnel/provision and medical facilities

Data extract (*n*)	Organising themes	Global themes
Unreliable medic provision company (4)	*Lack of reliability*	Reliability
Costs too great for team to have own medical staff (1)	*Medical costs*	Cost/funding
Overpriced medical cover (1)		
Home field playing ground varies across the season due to weather conditions (1)	*Lack of consistent grounds*	
AU responsible for paying and arranging medical cover (2)	*Lack of ownership over hiring of medical staff*	
Medical team with no experience of AF game (3)		
Medical bag organised by team physiotherapist. Unknown content of bag (1)		
Hiring of additional medical staff for away games (1)	*Going above the minimum medical recommendations*	
Coaching staff present with medical training for additional support (1)		
Request for AF specific first aid training if local and free (1)	*Willingness for first aid training*	Training
Limited or no access to field of play if an emergency (2)	*Access in emergencies*	Safeguarding risk
No risk assessment (1)	*Lack of sufficient emergency planning*	
Generic emergency action plan (1)		

## Discussion

This study expands upon prior study findings by Travis et al. [19] which highlighted players tendency to under-report injury, a lack of consistent medical provision and flaws within concussion safeguarding policy in BAF by further evaluating medical provision, facility and equipment access in the game.

Less than half of all participating teams reported access to a regular team first aider. The study reveals lack of consistent medical personnel, particularly at training sessions and away games, and training in emergency care, e.g. use of an AED. Yet, there remain a number of teams who report positive practice through support of their institution. The varying results of this study will need to be addressed by BAFA. The findings will be discussed in detail below.

### Medical personnel/provision

Appropriate medical provision is fundamental to the longevity of athletes’ playing careers and health when injuries occur [20, 21]. Like BAFA, other NGBs such as the Rugby Football Union (RFU) stipulate the level of medical cover required at games (including BUCS which differs across the league levels) [12, 13]. Previous research has indicated that higher leagues have access to more support staff [22]. However, this study found that there was no relationship between divisions and access to a regular team first aider suggesting that the division standing of a team has no impact on team finances or access to knowledgeable medical practitioners during games. The reason for the varied medical provision across the leagues might be explained by findings within the thematic analysis which highlighted institutional barriers as the key restriction to suitably qualified practitioners with BAF experience. One of these barriers was the cost of financing medical cover. As one participant reported, ‘the teams athletic union is responsible for paying for and arranging medical cover’, i.e. a sports therapist, paramedic, highlighting the lack of control in the selection of appropriate medical support. This statement was supported by another team who stated, ‘there’s little to support us receiving an experienced medical person to cover’. Access to medical provision for some teams is, therefore, out of the hands of the BAF BUCS team themselves who may be more aware of the intricacies of the type of medical provision that is required for BAF and so better placed to hire personnel.

Findings indicate that there is a shortage of suitable medical practitioners with knowledge of the game to cover events. Similar situations have been noted in Australia where it was reported that there was a shortage of doctors willing to cover sporting events due to inadequate remuneration, venue facilities, training opportunities in sports medicine and fear of medicolegal consequences [23]. Moreover, research in British mixed martial arts (MMAs) has found medical provision to be sacrificed in the pursuit of profit [24]. At present, it is unclear who is responsible for funding medical provision of BAF university teams; however, provision is likely organised and funded by the Athletic Union (AU) via student membership fees. Approach to hire could differ between universities. Some of these issues were raised in the qualitative comments on this survey. For example, costs were raised as a barrier to hiring suitably qualified medics. One team noted that they found them ‘unreliable’ and ‘the people they would send would have no experience or understanding of the game’. Another team noted that the ‘cost of trained professionals is a real roadblock’. However, one team had a more positive experience in hiring through an external company, whose medics were ‘suitably covered to cover away games’. This raises the question as to whether the participants fully understand what ‘suitably covered’ means. Indeed, research has found that appointments of medical staff in the Football Association (FA) and MMA are still informal and that continuous professional development (CPD) requirements are not prioritised despite the current medical regulations [25–27]. There is, therefore, reason to believe that across sports, there is either practice of ignorance or disregard for the regulations.

The majority of teams who did not have a regular team first aid practitioner, used a coach with first aid qualifications during training (54.8%), suggesting that many coaches are recruited with first aid qualifications or complete this training during their coaching season. However, the level of qualification they hold is unknown. The British American Football Coaches Association (BAFCA) at present does not require coaches to have first aid certification, yet coaches are key personnel to have trained in first aid because they have the most regular contact with players and have influence over practice and team culture in respect of injury [28]. However, the fact that coaches are relied upon to provide medical cover is not unique to BAF. Similar trends are seen in Irish Rugby Union and English Youth Football where the majority of first aiders are coaches or officials [29, 30], which possibly compromises the level of medical care provided due to the dual roles of these staff.

A second concern is the use of players with a first aid qualification as the primary first aider at training. Almost a third of teams reported that a player provided first aid cover at training. This is poor practice, as should that player become injured themselves, there is no-one to care for them. Worryingly, this places the ‘first aider/player’ with competing priorities, such as short-term performance gains, e.g. winning vs the athlete’s welfare [31] which could be more significant within the first aider/player role. The prior 2017 BAFA policy stated that a member of the team could not be named medical cover in a game. However, the policy did not stipulate this in a training environment, when it is likely this sub-standard practice was taking place. Positively, the updated 2022 guidance states that no player nor official can participate in training and act as medical cover [32].

Some university teams are in a unique position to draw upon the support of medical students. One team commented that they have a ‘student sports therapist who attends occasional Sundays and home games’. This is beneficial to both the student experience, the teams they are supporting and in also developing a group of practitioners with BAF game experience [33]. However, these students should work under the observation of qualified practitioners, who also hold suitable insurance to allow the student to practical under their guidance [34]. It is unclear whether this is currently occurring, or whether the students are practicing on their own; however, it is not uncommon in combat sports for unqualified practitioners to work at events [27]. Secondly, it is concerning that some teams reported utilising students in a training environment, where students currently in medical training might be expected to work outside of their current and insured scope of practice. It would be advisable for the NGB to advise against the use of a student as medical provision without the support of a suitably qualified professional.

### Facilities

Positively, the majority of clubs had access to an AED. The ability to resuscitate those in cardiac arrest is a key consideration as use of an AED can improve survival rates [35–37] and so is recommended to be present in every sports facility [38]. The majority of teams (74.2%) had access to an AED within 100 m of the game facility, yet only 51.6% listed an AED as available in their pitch-side first aid equipment and only 29% of staff were trained to use them. It is concerning that so few staff have training to use this equipment and only half of teams reported AED access as part of their first aid provisions. A 2009 review highlighted three areas that are critical to improving cardiac arrest survival: the presence of a trained rescuer to initiate CPR, early defibrillation, and access to on-site AEDs to initiate early defibrillation [39]. It is recognised in the commentary themes that inadequate funding is a barrier to suitable medical provision which might explain the limited pitch-side AED access.

It should be noted that innovative practice was observed within the commentary section of the survey. One team reported ‘all coaches also use the “what three words app”’, a tool designed to specify a very specific location, to support navigation in emergencies. This is a unique approach to game day practice, which is previously unknown to the researchers, yet recommendable to others to follow alongside current policy.

### Policy compliance

The level of compliance by BUCS teams is somewhat difficult to evaluate due to the role of the university AU in game day facilitation. Just over half of teams (67.7%, *n* = 21) had outlined an EAP for each home game, despite BAFA medical policy. However, the majority of teams (83.9%, *n* = 26) reported access to a designated fully charged mobile phone for game days, showing the majority of teams were compliant to only one element of BAFA medical policy in 2019/2020.

Nonetheless, it should also be noted that each individual BUCS team will be required to follow their institutions AU guidelines with respect to game-days, which further adds to the complexity of compliance. BAFA states that if their medical regulations are not met (as deemed by the game day referees), the game should be suspended or cancelled. This raises the question as to whether referees are sufficiently checking the medical provision prior to each game. League officials should look to drive best practice for player welfare as on occasions, officials may not ensure teams are meeting the minimum standards of compliance.

This survey found that 61.3% (*n* = 19) of the teams were unsure if the medical personnel had the required qualifications to meet the game-day minimum requirements. However, some teams may have been unaware of the first aiders qualifications despite the first aider being well qualified, particularly if medical cover is arranged through the AU. It appears that this is not unique: Coughlan et al. [30] found that many rugby union clubs were without acceptable provision for the level of competition. It could, thus, be argued that increasing the number of medical practitioners at BAF games is wise. Indeed, it has been questioned whether meeting the minimum medical standards is enough [38, 40]. Hence, revising the current medical guidelines for game day and training is advisable.

### Policies/procedures and documentations

Emergency action plans (EAPs) provide guidelines to support the management of emergencies including acute injuries [39], define the procedures to be used in the acute management of injury and provide a plan for mild through to life-threatening injuries of all involved in the game [41, 42]. EAPs should be specific to the facility, population, medical personnel and documentation [43]. A lack of defined emergency procedures can increase the time delay in emergency situations, providing a poorer chance of outcome for those injured [41]. The prior 2017 BAFA medical policy stated within the minimum requirements for games that the game day medic ‘must have carried out a risk assessment’ and ‘a telephone capable to use to summon the emergency services must be available’. Yet our data has highlighted inconsistent medical care; thus, it is not clear if each new medical practitioner is conducting a risk assessment prior to a game or if the club has written their own risk assessment and that the game day medical practitioners are aware of its details. Binder [41] highlighted that identification of medical personnel is critical to success of an EAP further highlighting the need for consistent practitioners to be present. Secondly, a risk assessment differs from an EAP. The risk assessment often briefly outlines injury risk but not the procedures to follow if an incident occurs.^2^ With 58.1% of teams reporting a lack of consistent medical cover, it could be presumed that teams lack EAPs and the practice simulation of emergency scenarios should an incident occur. This puts athletes at risk of further harm and the team staff at risk of negligence accusation due to a lack of their duty of care [41]. Simple steps can be taken through the development of emergency plans to both support and protect players and staff [41]. For example, the NGB could support the education of teams on EAP development, providing an example EAP for each team to revise for their own needs which could include step-by step plans for responding to minor and major emergencies [41].

Confidence, cohesion and trust in medical staff are important for both athletes and coaches [44–46]. In this survey, there was a common theme of lack of game knowledge by first aiders. Whilst confidence was not directly measured, it indicates that some teams may not have been wholly satisfied that the first aider knew about the game and common injuries in BAF. Research suggests that effective teams work in a multidisciplinary manner, using open communication and having clearly defined roles [47]. Teams without a clear and consistent medical provision plan cannot hope to manage player welfare and are unlikely able to build an effective performance environment [39, 48, 49]. In order to develop the sport in this country and increase participation, strong and stable club organisation is key to development [50].

### Limitations

Only 38% of BUCS teams participated in this survey. Therefore, these data might not be representative of all BUCS club medical provision. However, whilst the response rate was small, they were comparable to similar audits in rugby union [22, 30], and the results and opinions should not be discredited.

### Future directions

Medical polices and associated practices could be supported through educational workshops and training provided by the NGB. With recognised training and qualifications in place, the NGB could stipulate in policy guidelines that only accredited qualified medical practitioners can cover BAF games. However, the practicalities of this implementation should be evaluated and discussed with all stakeholders. Implementation of appropriate medical cover would ensure that teams are adequately supported, with the correct training to deal with medical emergencies relating to the sport of American Football. Any new programmes or guidance should be reviewed on the success and uptake to support continued delivery and improvement. Further suggested policy is stated in Table 4.

**Table 4:** Suggested policy

1.	Randomised checks of medical provision to take place to ensure policy is being followed, ensuring guarding player welfare. These checks could be conducted by senior referees since referees conduct checks on game day.
2.	At least one member of game day staff should have AED training. Teams should guarantee this staff member is present at every game day.
3.	No student may practice alone as a first aider during games or training. Students must be supervised by a qualified and insured practitioner.
4.	University team management should have a say in the hiring of medical practitioners and be aware of the qualifications of the medical practitioner.
5.	First aid training specific to AF to be made available to medical practitioners and coaching staff.
6.	A separate BUCS game medical policy to be written to allow for differences between the BUCS game and the adult game.
7.	Clarity to be provided within policy on whether the purpose of the first aider is for musculoskeletal injury support (e.g., athletic taping, injury assessment) or lifesaving.
8.	Direct emergency vehicle access to field of play is necessary.
9.	Training to be provided to all teams to ensure understanding of updated policy.
10.	An emergency action plan (EAP) should be outlined by each team for games and training which includes planning for minor and major incidences.
11.	The home team should provide access to a designated medical room, ensuring the possibility of private and confidential medical assessments or treatment in a sterile environment.

## Conclusions

This study provides key information on the medical provision, facilities and protocol compliance in BUCS BAF. The findings of this survey will allow the key stakeholders to identify gaps within the current medical provision to support the development of provision and implementation of educational programmes following the introduction of new medical provision guidance.

## Footnotes

1 At the time of data collection, the BAFA medical policy had not been updated since 2017 and it did not advise on medical requirements at training [11]

2 It should be noted that the new BAFA medical policy highlights the difference between risk assessments and EAPs, showing progress [32]

## Declarations

### Disclaimer

The views expressed in this study are not influenced by the committee.
